# The effect of the Thanksgiving Holiday on weight gain

**DOI:** 10.1186/1475-2891-5-29

**Published:** 2006-11-21

**Authors:** Holly R Hull, Duncan Radley, Mary K Dinger, David A Fields

**Affiliations:** 1 Department of Health and Exercise Science, University of Oklahoma, Norman, OK, USA; 2 Carnegie Research Institute, Leeds Metropolitan University, UK; 3 Department of Pediatrics, University of Oklahoma Health Science Center, Oklahoma City, OK, USA; 4 Children's Medical Research Institute's Metabolic Research Center, USA

## Abstract

**Background:**

More people than ever are considered obese and the resulting health problems are evident. These facts highlight the need for identification of critical time periods for weight gain. Therefore the purpose was to assess potential changes that occur in body weight during the Thanksgiving holiday break in college students.

**Methods:**

94 college students (23.0 ± 4.6 yrs, 72.1 ± 14.0 kg, 172.6 ± 9.3 cm, 24.0 ± 3.9 kg/m^2^) reported to the human body composition laboratory at the University of Oklahoma following a 6-hour fast with testing occurring prior to, and immediately following the Thanksgiving holiday break (13 ± 3 days). Body weight (BW) was assessed using a balance beam scale while participants were dressed in minimal clothing. Paired t-tests were used to assess changes in BW pre and post Thanksgiving holiday with additional analysis by gender, body mass index (BMI), and class standing (i.e. undergraduate vs. graduate).

**Results:**

Overall, a significant (*P *< 0.05) increase in BW was found between pre (72.1 kg) and post (72.6 kg) Thanksgiving holiday. When stratified by gender and class standing a significant (*P *< 0.05) increase in body weight was observed between the pre and post Thanksgiving holiday in males (0.6 kg), females (0.4 kg) and graduate students (0.8 kg). When participants were classified by BMI as normal or as overweight/obese, a significant 1.0 kg BW gain was found (*P *< 0.05) in the overweight/obese (≥25 kg/m^2^) group compared to a non significant 0.2 kg gain in the normal group (<25 kg/m^2^).

**Conclusion:**

These data indicate that participants in our study gained a significant amount of BW (0.5 kg) during the Thanksgiving holiday. While an increase in BW of half a kilogram may not be cause for alarm, the increase could have potential long-term health consequences if participants retained this weight gain throughout the college year. Additionally, because the overweight/obese participants gained the greatest amount of BW, this group may be at increased risk for weight gain and further obesity development during the holiday season.

## Background

In the United States 1 in 5 college students are classified as obese [1]. Perhaps more alarming is that previous research indicates obesity rates rising fastest in 18 to 29 year olds and those with some college education [2]. These are grave statistics given that for the first time in history, predictions suggest the youth of today may live shorter lives than their parents [3].

Obesity levels in adults are commonly attributed to a small prolonged discrepancy in energy intake and/or energy expenditure that results in a gradual yearly weight gain. In young adults this has been estimated at approximately 0.2 to 0.8 kg per year [4]. However, there is little available evidence to identify whether annual body weight increases are the resultant effect of the perceived continual daily discrepancy in energy balance or are due to more discrete periods of weight gain such as holiday periods. The holiday season is a time of the year suggested to present an increased risk of weight gain and obesity development [5]. This is postulated to be caused by stress associated with the holidays, increased caloric intake, and/or a decline in physical activity [6].

College students are a group of particular interest considering their reported weight gain during their freshman year. While the widespread campus perception that freshman students gain an average of 15 pounds (6.8 kg) during their first year of study appears little more than a myth, recent studies have still reported weight gains of 1.3 kg (Hoffman et al. 2006), 2.5 kg (Racette et al. 2005) and 1.1 kg (Morrow et al. 2006) [7-9]. Given this magnitude of weight gain is still considerable, it is important to understand the role of the Thanksgiving holiday period. To our knowledge, no study has examined weight changes over the Thanksgiving holiday in college students. Therefore, the aim of this study was to assess the changes in body weight over the Thanksgiving holiday in college students and to evaluate possible differences based on gender, body mass index, and class standing.

## Research Methods and Procedures

Participants were male and female students enrolled at the University of Oklahoma and were recruited via a mass email and announcements in college courses following approval by the University of Oklahoma Institution Review Board. Both undergraduate and graduate students were encouraged to participate. Participants were healthy and free from any disease known to affect metabolism or body weight. An informed consent was signed by each participant prior to the start of the study.

Participants visited the human body composition laboratory the week prior to the Thanksgiving holiday break and then returned 5 to 7 days following the holiday break. The days between the first and second visit were as follows: the minimum days between visits were 5 days, the maximum days between visits were 17 days and the mean and standard deviations for the group was 13 ± 3 days. During each visit, demographic data for each participant was obtained and body weight was measured using the Detecto Manual Physician scale while participants were dressed in light clothing (i.e. no shoes, sweaters, jackets, or belts) and height was assessed using a stadiometer (Accu-Hite Wall Stadiometer, Seca Corp., Hanover, MD) with shoes removed. A plastic flexible Gulick tape measure was used to assess waist and hip circumferences. The waist measurement was assessed at half the distance between the bottom of the xiphoid process and the umbilicus, and the hip measurement was taken at the largest anterior protrusion. The ratio between waist and hip was computed. All testing was completed with subjects fasted for 6 hours from food (i.e. no solid food or liquids) and physical activity.

Statistical analysis was performed using Statistical Package for Social Sciences (SPSS version 11.5). The means and the standard deviations of body weight and anthropometric variables were calculated. Paired t-tests were completed to assess if differences existed between visits for body weight and further analysis was completed with participants categorized based on gender, class standing (undergraduate vs. graduate) and BMI (Normal <25 kg/m^2^, Overweight/obese ≥ 25 kg/m^2^). Pearson correlation coefficients were calculated to assess the relationship between baseline BMI and weight change between visits. To examine the impact of the range of testing dates for the different groups, independent t-tests were completed. No differences were found (*P *> 0.05) for days between visits for any of the groups. Statistical significance was set at *P *≤ 0.05.

## Results

Of the 100 participants who attended the pre-Thanksgiving visit, 94 returned for the post-Thanksgiving assessment. Therefore, all analyses were conducted using data from the 94 participants (male = 44, female = 50) who completed both visits. Baseline characteristics of completers (N = 94) and non-completers (N = 6) were compared and no differences between the groups were found for any of the outcome variables measured (*P *> 0.05). Descriptive characteristics of the 94 participants are presented in Table 1. Participants were predominantly Caucasian (75%) but also included African Americans (5%), Asians (4%), Hispanics (10%) and Native Americans (6%).

**Table 1:** Descriptive characteristics of participants.

	Male (N = 44)	Female (N = 50)	Group (N = 94)
Age	24.4 ± 4.9	21.8 ± 4.1	23.0 ± 4.6
Height (cm)	179.7 ± 6.3	166.4 ± 6.7	172.6 ± 9.3
Weight (kg)	80.3 ± 12.5	64.9 ± 11.1	72.1 ± 14.0
BMI (kg/m^2^)	24.8 ± 3.4	23.5 ± 3.8	24.0 ± 3.9
Waist (cm)	84.6 ± 8.7	75.1 ± 9.9	79.6 ± 10.5
Hip (cm)	101.4 ± 8.1	99.2 ± 9.5	100.2 ± 8.9
Waist hip ratio	0.83 ± 0.04	0.76 ± 0.07	0.80 ± 0.07

Means ± Standard deviation

There was a significant increase in body weight (BW) between visit 1 (pre-Thanksgiving) and visit 2 (post-Thanksgiving) for the entire group (72.1 kg vs. 72.6 kg; *P *< 0.05) and when the group was classified as male (80.2 kg vs. 80.8 kg; *P *< 0.05) and female (65.0 kg vs. 65.4 kg; *P *< 0.05) (Table 3). When participants were classified based on class standing (undergraduate vs. graduate) graduate students increased body weight between visits (76.3 kg vs. 77.1 kg; *P *< 0.05), while no significant increase was observed for undergraduates (70.3 kg vs. 70.7 kg; *P *= 0.07).

**Table 3:** Body Weight changes by gender, class standing and BMI.

	Body Weight (kg)		
	
	Pre-Thanksgiving	Post-Thanksgiving	Δ pre-post
Group (N = 94)	72.1 ± 14.0	72.6 ± 14.3*	0.5 ± 1.5
Males (N = 44)	80.2 ± 12.5	80.8 ± 12.7*	0.6 ± 1.9
Females (N = 50)	65.0 ± 11.1	65.4 ± 11.6*	0.4 ± 1.0
Undergraduates (N = 66)	70.3 ± 13.4	70.7 ± 13.7	0.4 ± 1.7
Graduates (N = 28)	76.3 ± 14.8	77.1 ± 15.0*	0.8 ± 0.8
Normal BMI (N = 60)	64.7 ± 9.3	64.9 ± 9.4	0.2 ± 1.6
Overweight/Obese BMI (N = 34)	85.2 ± 11.0	86.2 ± 11.1*	1.0 ± 1.1

Means ± Standard deviations

*Significantly different from pre-Thanksgiving visit (*P *< 0.05)

BMI Categories: Normal <25 kg/m^2 ^and Overweight/obese ≥ 25 kg/m^2^

Class Standing: Undergraduate and Graduate

To attempt to assess changes in fat patterning, waist circumference, hip circumference and waist/hip ratio was measured. A significant (*P *< 0.05) decline in waist circumference was found for the group, males, females, undergraduates and normal BMI participants. Similar declines were also found in waist/hip ratio for males, females, undergraduates and normal BMI participants. A summary of these values are listed in Table 2.

**Table 2:** Changes in waist circumference, hip circumference and waist/hip ratio for the entire sample and by gender, class standing and BMI.

	Δ Waist Circumference (cm)	Δ Hip Circumference (cm)	Δ Waist/Hip Ratio
Group (N = 94)	-1.14 ± 3.5*	0.54 ± 4.0	-0.02 ± 0.04
Males (N = 44)	-0.98 ± 3.0*	0.75 ± 3.0	-0.02 ± 0.04*
Females (N = 50)	-1.29 ± 3.8*	0.36 ± 4.8	-0.02 ± 0.04*
Undergraduates (N = 66)	-1.41 ± 3.5*	0.13 ± 3.9	-0.02 ± 0.04*
Graduates (N = 28)	-0.51 ± 3.4	1.50 ± 4.2	-0.02 ± 0.04
Normal BMI (N = 60)	-1.25 ± 3.5*	0.84 ± 4.4	-0.02 ± 0.04*
Overweight/Obese BMI (N = 34)	-0.96 ± 3.5	0.02 ± 3.2	-0.01 ± 0.03

Means ± Standard deviation

*Significant difference between pre-Thanksgiving and post-Thanksgiving visits (*P *< 0.05)

BMI Categories: Normal <25 kg/m^2 ^and Overweight/obese ≥ 25 kg/m^2^

Class Standing: Undergraduate and Graduate

Additionally, participants were categorized based on BMI to determine if a BMI classification of normal (<25 kg/m^2^) vs. overweight/obese (≥ 25 kg/m^2^) impacted the change in body weight. No significant increase in body weight was found in normal BMI participants (64.7 kg vs. 64.9 kg; *P *= 0.27); however, overweight/obese participants increased body weight significantly (85.2 kg vs. 86.2 kg; *P *< 0.05). A summary of these results are listed in Table 3.

**Figure 1:**
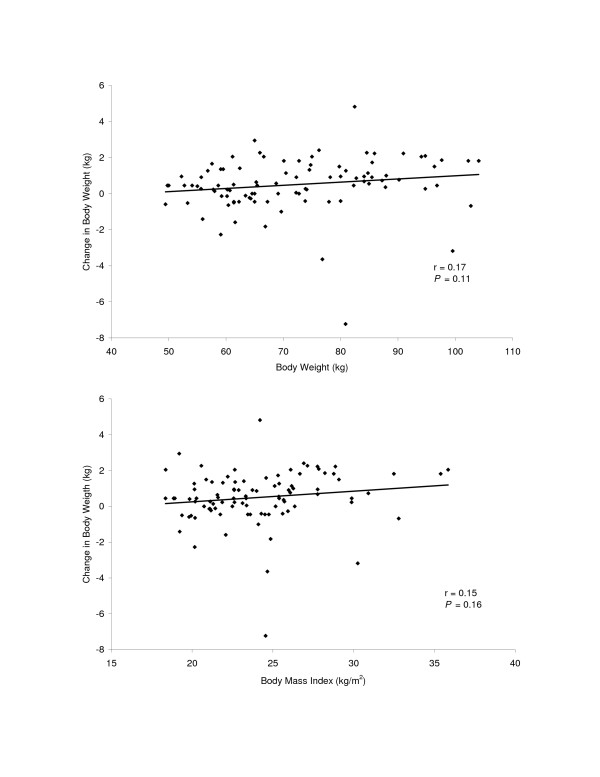
The top panel depicts the relationship between body weightat the pre-Thanksgiving visit to the change in body weight between the pre-Thanksgiving to the post-Thanksgiving visits. The bottom panel depicts the relationship between body mass index at the pre-Thanksgiving visit to the change in body weight between the pre-Thanksgiving to the post-Thanksgiving visits.

Correlations were calculated to assess the impact of body weight (r = 0.17, *P *= 0.11) and BMI (r = 0.15, *P *= 0.16) from the pre-Thanksgiving visit on changes in body weight. No significant correlations were found (Figure 1). Additionally, correlations were calculated to determine if baseline BMI was related to the weight change between visit 1 and visit 2. In females, a significant correlation (r = 0.42; *P *< 0.01) was found between baseline BMI and weight change between visits however no significant correlation was observed in males (r = -0.03; *P *= 0.43).

## Discussion

An increase in body weight was observed over the Thanksgiving holiday with males and females exhibiting similar trends (0.6 kg and 0.4 kg, respectively), however, the greatest increases in body weight were witnessed in graduate students (0.8 kg) and overweight/obese participants (1.0 kg).

Although a great deal of publicity is given to holiday weight gain, few research studies have been done to examine weight changes during the holiday season and only one research study was performed in college students [4,10-12]. Two studies have examined body weight changes that occurred over the entire holiday season: pre-Thanksgiving to post-New Year's. Yanovski et al. studied 195 adults and found the holiday season resulted in a significant (*P *< 0.001) increase in body weight of 0.37 kg [4]. Baker et al. examined weight changes during holiday compared to non-holiday weeks, over the entire holiday season, in a group of participants enrolled in an obesity prevention program. Participants gained 500% more weight (specific increase in body weight not reported) during the holiday weeks versus the non-holiday weeks [12].

Other research has specifically focused on either Thanksgiving or Christmas during the holiday season. Reid and Hackett studied 26 participants over the Christmas holiday and found an increase in body weight of 0.93 kg though the results may be confounded by a small number of participants and five participants reporting being ill during data collection [10]. A second study examined the effect of the Thanksgiving weekend on body weight in college students by assessing dietary records before, during and after the holiday [11]. They found significantly increased dietary intake over the Thanksgiving holiday with males consuming more calories than females and non obese consumed more calories than obese.

The holiday season is a time when cultural and social influences combine to create a high risk environment conducive to weight gain. A number of factors particularly prevalent during the holiday celebrations that encourage over consumption include: longer eating durations, easy access to food, eating in the presence of others and increase portion sizes [13]. De Castro (1995) reported that meals eaten in the presence of others were 44% larger than when meals were eaten alone, while Rolls et al. (2002) and Levitsky 2002 demonstrated that increased portion size is associated with increased consumption [14-16]. Given these factors it is hardly surprising that Drapkin et al. (1995) reported than out of four hypothetical high risk situations participants perceived a family mealtime celebration (which includes a holiday meal) as the time they would be most likely to overeat [17]. Further, Boutelle (1999) reported that both their intervention and comparison groups found it difficult to effectively manage their weight during the Christmas to New Year holiday period [5].

Several limitations to the current study should be noted. First, since participants were derived from a volunteer convenience sample a self-selection bias may have occurred such that subjects had a personal interest in monitoring their weight. Second, participants were aware the primary aim of the study was to investigate changes in body weight during the Thanksgiving period. This may have resulted in participants intentionally or unintentionally altering their patterns of behavior, consequently masking true changes in their body weight during the measurement period. In addition, participants may have intentionally tried to reduce their body weight in the days preceding their second measurement. Third, no information is available on changes in body weight during the periods preceding or following the Thanksgiving holiday.

In conclusion, we found over the Thanksgiving holiday an increase of 0.5 kg in body weight. Although this may seem like a trivial amount of weight, considering the short time frame, this is troublesome since previous research suggests weight gained during holiday periods is retained (Yanovski 2000). Therefore, we found in our sample, the Thanksgiving holiday represented a critical period for weight gain and obesity development. Additionally, it seems as though graduate students or those who are already overweight/obese are at increased risk of greater weight gain. These findings may have important practical implications given the need for implementation of effective intervention strategies in those groups most at risk for obesity development and its associated co-morbidities.

## Competing interests

The author(s) declare that they have no competing interests.

## Authors' contributions

HH and DF conceived the study and wrote the manuscript while HH collected the data. MD and DR provided critical review and revisions to the manuscript.
